# Multi-classification method of arrhythmia based on multi-scale residual neural network and multi-channel data fusion

**DOI:** 10.3389/fphys.2023.1253907

**Published:** 2023-09-28

**Authors:** Fuchun Zhang, Meng Li, Li Song, Liang Wu, Baiyang Wang

**Affiliations:** ^1^ School of Information Science and Engineering, Linyi University, Linyi, China; ^2^ School of Radiology, Shandong First Medical University & Shandong Academy of Medical Sciences, Tai’an, China; ^3^ School of Information Science and Engineering, Shandong University, Qingdao, China

**Keywords:** deep learning, multi-scale, residual neural networks, electrocardiogram, multichannel fusion

## Abstract

Since ECG contains key characteristic information of arrhythmias, extracting this information is crucial for identifying arrhythmias. Based on this, in order to effectively extract ECG data features and realize automatic detection of arrhythmia, a multi-classification method of arrhythmia based on multi-scale residual neural network and multi-channel data fusion is proposed. First, the features of single-lead ECG signals are extracted and converted into two-dimensional images, and the feature data sets are labeled and divided according to different types of arrhythmias. The improved residual neural network is trained on the training set to obtain the classification model of the neural network. Finally, the classification model is applied to the automatic detection of arrhythmias during exercise. The accuracy of the classification model of this method is as high as 99.60%, and it has high accuracy and generalization ability. The automatic identification of arrhythmia also contributes to the research and development of future wearable devices.

## 1 Introduction

Data released by the American College of Cardiology in 2019 shows that cardio-vascular disease is already the most important cause of human death. It is estimated that by 2030, the number of deaths from cardiovascular disease will reach 24 million (Sannino and De Pietro, 2018; Liu et al., 2021). Arrhythmias are a type of cardiovascular disease, and arrhythmias are usually not life-threatening, but some arrhythmias are harmful and can lead to stroke or sudden death (Luo et al., 2017). With the increase of social pressure, people often maintain physical health through exercise, and arrhythmias during exercise can lead to physical discomfort, and serious ones can also lead to sudden death. Electrocardiogram (ECG) is able to reflect the condition of the heart by recording periodic changes in the heartbeat (Fan et al., 2021). Therefore, arrhythmias can be diagnosed by electrocardiogram. It can be seen that it is very necessary to detect arrhythmic diseases as soon as possible through abnormal electrocardiograms, so as to achieve early intervention and early treatment.

The ECG signal is constantly changing with the beating of the heart, and it is a periodic repetition of heartbeat activity over time. Cardiologists detect abnormal heart-beats based on changes in the frequency, rhythm, and morphology of the heart-beat. The ECG signal corresponding to arrhythmias is short-lasting and imperceptible, which makes it easy for doctors to make mistakes in the diagnosis process, and the diagnosis process is time-consuming and laborious (Yang and Shen, 2013; Atal and Singh, 2020).

In order to achieve automatic classification of ECGs, researchers have proposed many methods (Cortes and Vapnik, 1995; Kleinbaum et al., 2002; TENCON, 2013; Xu et al., 2018), including artificial neural networks, support vector machines, logistic regression, and random forests. In recent years, deep learning technology has been used more and more widely in ECG. Based on this, we have a new understanding of the classification of ECG, and the deep learning-based method does not require additional feature extraction and selection steps (Breiman, 2001; Xu and Liu, 2020). Neural networks are able to ex-tract representative features from the input data and classify them with the help of softmax. Panda R et al. (Panda et al., 2020) proposed a new method to detect shockable ventricular cardiac arrhythmias using ECG signals. A fixed frequency range empirical wavelet transform filter bank is proposed for multi-scale analysis of ECG signals. Patterns evaluated using a fixed frequency range empirical wavelet transform of ECG signals were used as input to a deep convolutional neural network for the detection of shockable ventricular cardiac arrhythmias. Zhao W et al. (Zhao et al., 2019) based on the ResNet model, using a low-pass filter to eliminate noise in the ECG signal, obtained high classification accuracy on 5 different types of ECGs. Shi Z et al. (Shi et al., 2021) improved the residual network to achieve the 5-classification task of arrhythmias, and their classification accuracy reached 99.59%. Park J et al. (Park et al., 2020) designed the residual network structure of the integrated squeeze-and-excitation module and obtained 97.05% accuracy. Li D et al. (Li et al., 2020) proposed a multi-scale residual network and realized the 4-classification task of ECG signals, and the accuracy of the method reached up to 94.29% (Guo et al., 2021). combined multiscale and squeeze-and-excitation modules for arrhythmia classification, which extracted ECG image features and applied key features to signal recognition, and they verified the effectiveness of the method in two datasets with an accuracy rate of 98.3% and 97.5%, respectively (Mangathayaru et al., 2021). evaluated the diagnostic model using a single-lead dataset, where they first extracted the characteristics of the signal using the wavelet transform, and then used the attention mechanism to distinguish different arrhythmia categories from the extracted signal according to the time series, with an accuracy rate of 98.5%. In the above work, the ResNet model was used, the improved residual network and the fusion multi-scale residual network were used to realize the multi-classification task of arrhythmia. Their recognition accuracy has reached more than 90%, but in the medical field, the recognition of diseases is accurate the rate is closely related to the patient’s condition. Therefore, it is very important to further improve the recognition accuracy of ECG signals. Moreover, on the basis of obtaining better signal recognition accuracy in existing research, it is necessary to simplify the process of signal feature extraction, and the fusion of multi-lead data should also be considered. The data of different leads has different characteristics, and the data set composed of data extracted from different leads is more complex and confusing. The model trained in this way can be used in the face of complex and diverse clinical conditions. More accurately identify the disease.

Therefore, on the basis of the above research, drawing on the excellent algorithms in the field of im-ages, we propose a method based on multi-scale residual neural network and multi-channel fusion for the diagnosis of motor arrhythmias. The main contributions of this paper are as follows.

(1) The two-channel ECG data is fused and converted into a two-dimensional ECG image.
(2) A multi-scale module is integrated in the bottom layer of the residual network to fuse different scale features of ECG images. When the image features reach the deep layer of the network, the image information of the upper layer and the bottom layer can be combined through the multi-scale module.
(3) The improved network makes full use of image information of different scales, combines more image information to learn more content, alleviates the gradient disappearance that may occur in the deep network, and achieves higher-precision ECG classification.

The rest of this paper is organized as follows. Experimental materials and methods are described in the second section, including datasets, data pre-processing, im-proved residual neural networks and experimental equipment. The experimental results are described in Section 3 and discussed in Section 4. A summary of our work and vision for the future are described in section 5.

## 2 Materials and methods

### 2.1 Dataset

**FIGURE 1:**
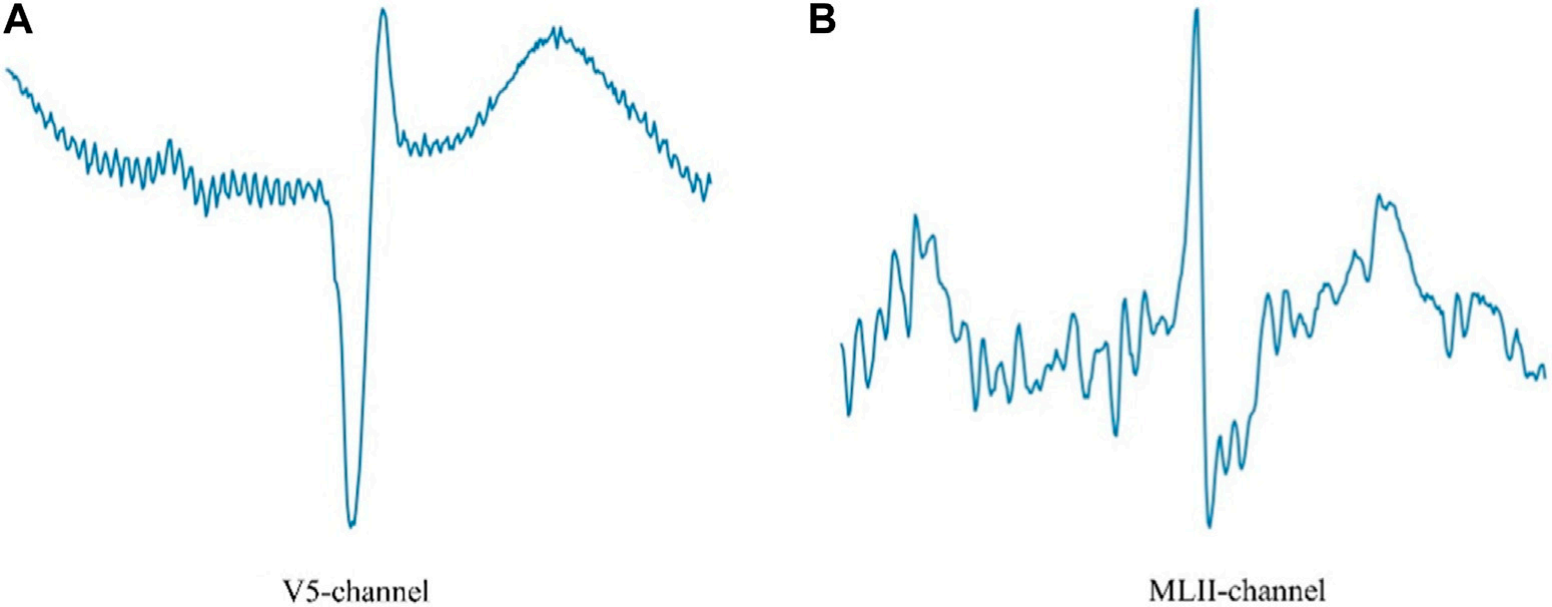
V5 lead and MLII lead ECG.

We completed the experiment using the MIT-BIH arrhythmia dataset (Goldberger et al., 2000; Moody and Mark, 2001). The heartbeat data recorded in this dataset includes two sets of leads. In Figure 1, A represents the V5 lead and B represents the MLII lead. This dataset randomly selected 23 cases from 4,000 24-h Holter records as representative samples of clinical routine records.

The sudden cardiac death holter dataset is data on sudden cardiac deaths recorded at boston hospitals in the 1980s. It included 18 patients with underlying sinus rhythm, 1 patient with continuous pacing, and 4 patients with atrial fibrillation. Annotating this dataset is particularly difficult due to the complexity of the heart rhythms included, so there is limited annotated data for this data.

#### 2.1.1 Dataset division

Deep learning algorithms tend to learn categories with a larger number of samples, and the number of ECGs for each arrhythmia in the MIT-BIH database is very different, so this will reduce the accuracy of the deep learning model. Therefore, we train the model by selecting categories with an approximate number of samples from the da-ta-base, which eliminates the impact of database imbalances. We selected 5 different categories from our database, including normal (N), left bundle branch block (LBBB), ventricular premature beats (PVC), atrial premature beats (APB), and right bundle branch block (RBBB).

The experimental dataset is divided according to the ratio of 9:1, and the number of MIT-BIH database ECG image samples used during training and testing is shown in Table 1. Table 1 shows the size of the sample 2D ECG dataset used in this paper. Among them, the number of ECGs for each arrhythmia category is also relatively close, so when training, the phenomenon of unbalanced model training can be avoided.

**TABLE 1:** Number of samples of MIT-BIH database used in training and testing.

Classes	Training set	Testing set	Total
N	9,252	1,027	10,279
LBBB	7,244	804	8,048
PVC	5,481	609	6,090
APB	2,291	254	2,545
RBBB	4,842	537	5,379

The number of samples in the sudden cardiac death holter dataset (Greenwald et al., 1985; Greenwald, 1986; Goldberger et al., 1988; Courtemanche et al., 1989; Goldberger et al., 1989) is shown in Table 2. The dataset is divided into three categories, namely, normal (N), ventricular premature beats (PVC) and Isolated QRS-like artifact (I).

**TABLE 2:** Number of samples of sudden cardiac death holter dataset used in training and testing.

Classes	Training set	Testing set	Total
N	9,839	1,093	10,932
PVC	9,626	1,069	10,695
I	9,526	1,091	10,617

### 2.2 Data pre-processing

This paper uses the matplotlib library of the python language to fuse the single-lead ECG data together, and uses the plt function to save it as a two-dimensional ECG image suitable for the CNN structure. Since each ECG signal is a continuous one-dimensional time series, we use 400 data points as a sample, data 1–400 as the first sample generated by cutting, and the starting point of the next sample is 401–800, until the end of ECG data.

$$S\times P+1\leq d\leq S\times P+P\;S\in \left(0,1,2,\ldots ,\frac{N}{P}\right)$$ (1)

The definition of each sample is shown in Equation 1, d is the current data point, P is the interval length of the selected data point, and S is the sample data after segmentation.

**FIGURE 2:**
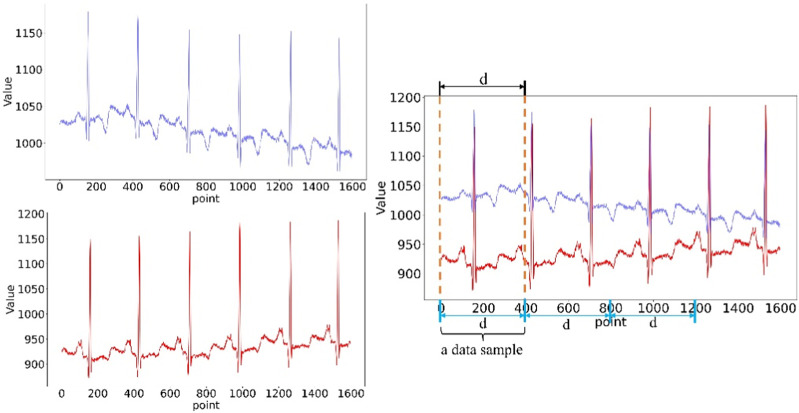
Data pre-processing.

The segmented data points are shown in Figure 2, with a sample of every 400 data points and looped through them.

### 2.3 Cross-entropy loss function

The function of the loss function is to calculate the gap between the predicted value of the network model and the true label, and it is used to measure how well the network model is trained. As the network model is trained, the loss function value changes continuously. When the loss function reaches the minimum value, the prediction accuracy of the network model also reaches the highest value. The cross-entropy loss function is used for multiclassification tasks, which can calculate the degree of difference in the probability distribution of the same random variable. Dataset D = ( $x_{1},y_{1}$ ), ( $x_{2},y_{2}$ ), … ,( $x_{N},y_{N}$ ), $x\in {\left\{x_{i}\right\}}_{i=1}^{N}\in X$ is the input variable, N represents the total number of samples, $y\in {\left\{y_{i}\right\}}_{i=1}^{N}\in Y$ is the predicted value of the expected output, this work is a multi-classification task, *y* ∈{1,2,…,K}, K is the number of categories. The cross-entropy loss function is defined as Equation 2:

$$L=\frac{1}{n}\sum_{n=1}^{N}\sum_{k=1}^{K}y_{nk}\log\;\left({\hat{y}}_{nk}\right)$$ (2)

### 2.4 Improved residual network

HE K et al. (He et al., 2016) proposed the residual neural network, which achieved success in the ImageNet competition. But considering that image features at different scales are also critical for the classification of ECGs, we made further improvements to the residual network.

In this paper, an improved multi-scale residual ECG classification algorithm is introduced, the network architecture is based on the residual structure, and takes into account the extraction of feature information at different scales, in order to make effective use of this information, multi-scale modules are added to the end of the residual network.

**FIGURE 3:**
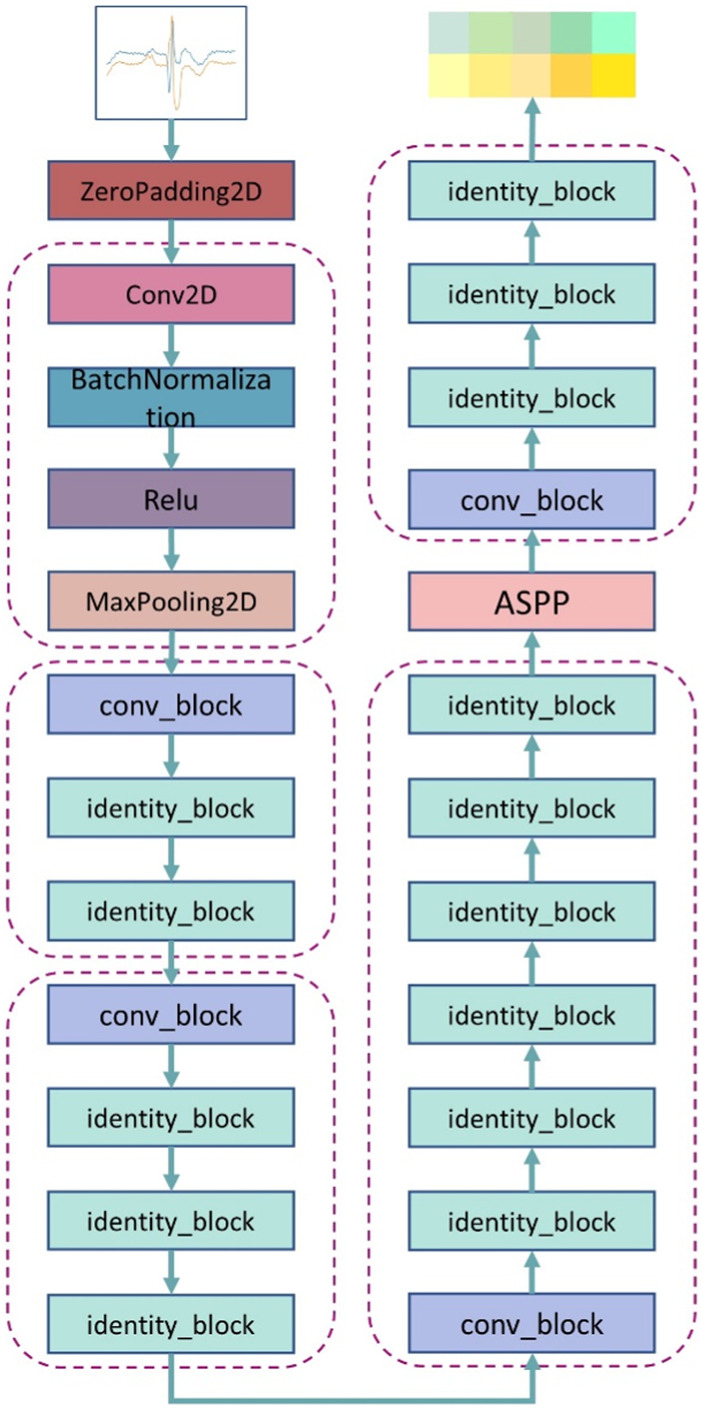
The overall architecture of a multiscale residual neural network.

The proposed network framework is shown in Figure 3, from which each module of the network architecture can be clearly seen. First, the 2D ECG signal is input into the network, and then it is zeroed, followed by 2D convolution and regularization operations. Before the residual connection, perform the maximum pooling operation, save the main features in the obtained feature map, and then input the main features into the residual block. We define two residual blocks, conv_block (denoted by ConvB below) and identity_block (denoted by IdenB below). First, the features obtained by the maximum pooling are input into the ConvB module, and then the output of the ConvB module is passed to the IdenB module, so that the obtained features retain the most important information as much as possible, so that the IdenB module is used more than the ConvB module. Because the IdenB module directly connects the input features with the final convolution output features, the ConvB module does not simply connect the initial input features, but performs convolution and batch regularization operations to further process the extracted features. The IdenB module receives the output of the ConvB module, and continuously retains the features of the initial acquisition to the subsequent convolutional layer, which can effectively preserve the features of the initial acquisition image and is more conducive to image classification. We add 2 IdenB modules after the ConvB module, 3 IdenB modules after the ConvB module, and 5 IdenB modules after the ConvB module, and fuse the multi-scale modules at the output position of this layer. As the network deepens, the training accuracy of the model is improved, but with continuous training, the gradient in the network will gradually disappear. When the network reaches a certain depth, a multi-scale module is added to sample the collected features at different sampling rates, so that the network can consider feature information of different scales, thereby improving the accuracy of classification.

#### 2.4.1 Conv_block module

**FIGURE 4:**
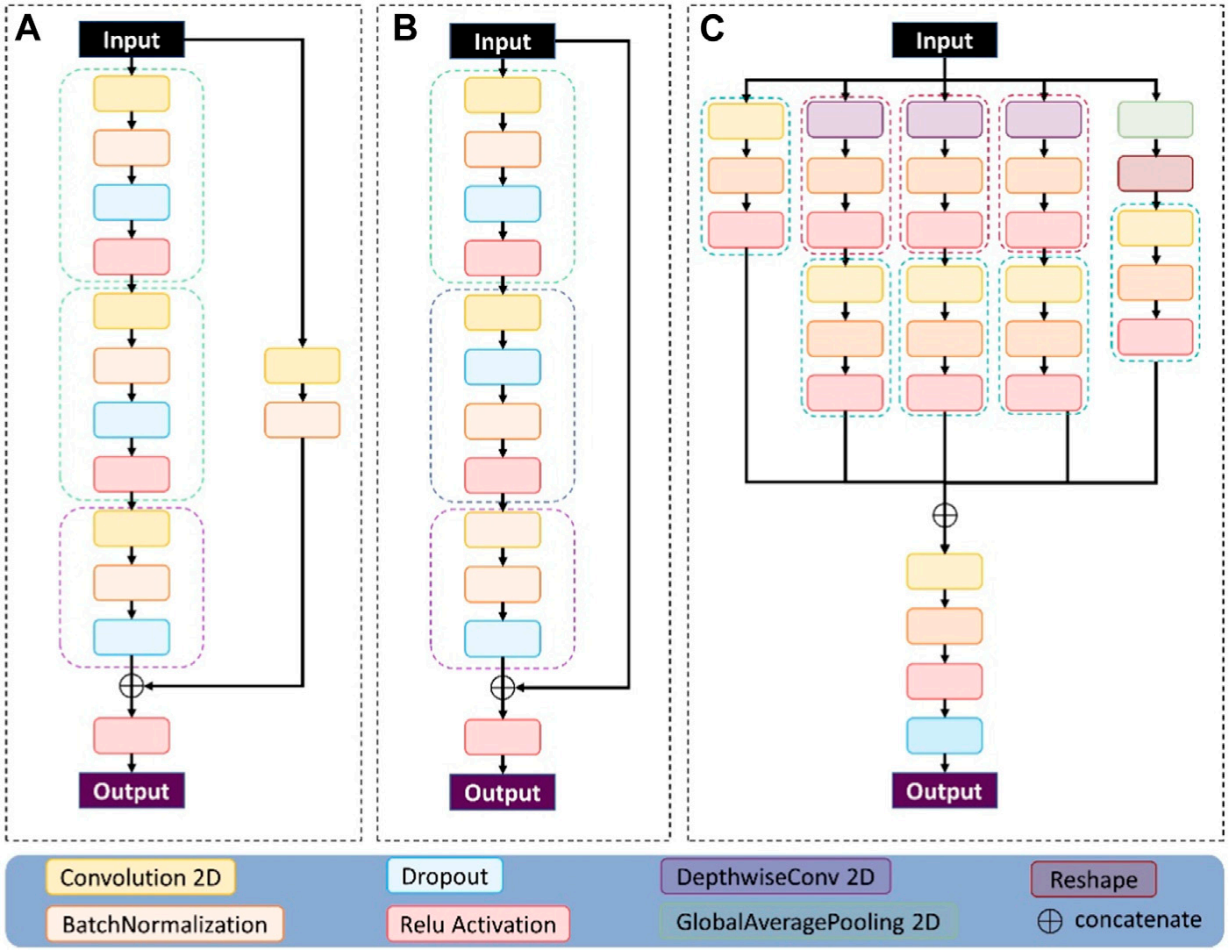
The three modules used in the network structure proposed here: **(A)** represents the conv_block module, **(B)** represents the identity_block module, and **(C)** represents the multi-scale module.

The contents of the ConvB module are described in this section, as shown in Figure 4A. The imported features are activated by two-dimensional convolution, batch regularization, dropout, and Relu in turn, and after four rounds of these four operations, the original input features are subjected to two-dimensional convolution and batch regularization operations, that is, shortcut. Connect the features of the output after four rounds of operation with the features of the output after the shortcut, and finally activate the output using a Relu.

#### 2.4.2 Identity_block module

The contents of the IdenB module are described in this section, as shown in Figure 4B. The imported features are in turn two-dimensional convolution, batch regularization, dropout, and Relu activation, and these four operations use the characteristics of the output after three rounds to connect the original input features, and finally use a Relu to activate the output.

#### 2.4.3 Multi-scale module

### 2.5 Diagnostic methods

**FIGURE 5:**
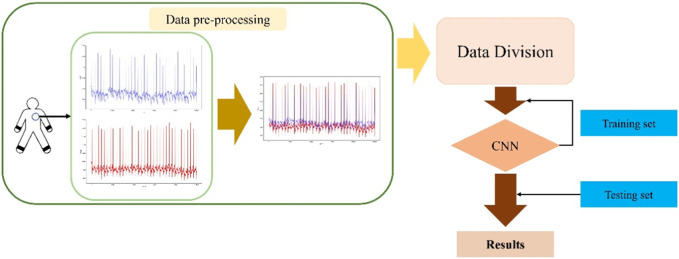
Flowchart of diagnostic methods.

The method flow mainly includes: data preprocessing, dataset division, training model and ECG image classification. Figure 5 shows the flow of the method presented herein.

1. First collect ECG data, which is one-dimensional time series data;
2. Preprocess the collected 1D ECG data. The single-channel data was fused by the matplotlib method to form a two-dimensional multi-channel ECG image;
3. In a 9:1 ratio, the ECG dataset is divided into training and validation sets;
4. In order to fully extract the characteristics of multi-channel ECG images, multi-scale network modules are designed;
5. Train the designed model to obtain the network model of ECG image diagnosis;
6. The trained model is verified on the validation set and the accuracy of ECG image recognition is calculated.

This paper uses the frameworks of Python and Tensorflow to complete the experiment. The program was trained and tested with the GPU NVIDIA GeForce RTX 3060. During training, in order to reduce the memory usage, each group takes 32 pieces of data, and the learning rate of the Adam optimizer is 0.0001.

## 3 Results

$$\text{Acc}=\frac{\text{TP}+\text{TN}}{\text{TP}+\text{TN}+\text{FP}+\text{FN}}$$ (3)

The definition of the evaluation index of the experimental results is as follows:

$$\text{Precision}=\frac{\text{TP}}{\text{TP}+\text{FP}}$$ (4)

$$\text{Sensitivity}=\frac{\text{TP}}{\text{TP}+\text{FN}}$$ (5)

$$F1\text{ score}=\frac{2\cdot \text{Precision}\cdot \text{Sensitivity}}{\text{Precision}+\text{Sensitivity}}$$ (6)

TP is to predict a positive class as a positive class, FN is to predict a positive class as a negative class, FP is to predict a negative class as a positive class, and TN is to predict a negative class to be a negative class.

**FIGURE 6:**
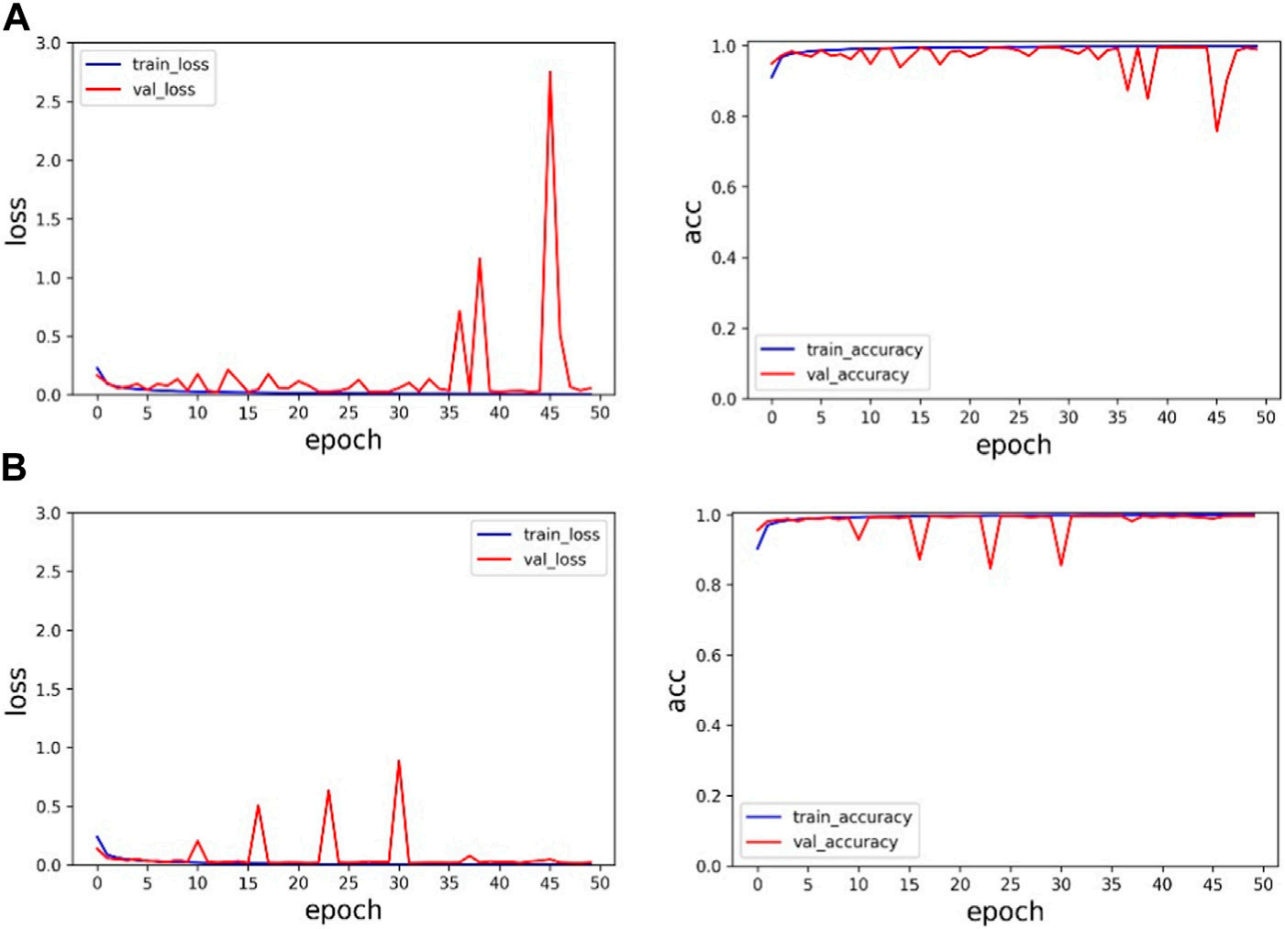
Experimental results on the MIT-BIH dataset: **(A)** is the experimental result of ResNet, and **(B)** is the experimental result of the proposed method.

Firstly, in order to verify the importance of multiscale modules, the experimental results of ResNet and the proposed method are compared under the same dataset. The training results are shown in Table 3 and Figure 6. Table 3 summarizes the experimental results under the same dataset, and the accuracy of ResNet and the proposed method in the verification set is 98.99% and 99.60%, respectively. The accuracy of the proposed method on the validation set is 0.61% higher than that of ResNet, and the loss function value on both the training set and the validation set is lower than that of ResNet. Therefore, it is very reasonable to add multi-scale modules on the basis of ResNet, and multi-scale modules can effectively improve classification accuracy.

**TABLE 3:** Loss values and acc in the MIT-BIH dataset.

Methods	Train_loss	Val_loss	Train_acc	Val_acc
ResNet	0.003	0.05	99.86	98.99
Proposed	0.003	0.02	99.91	99.60

In Figure 6, the first line represents the loss function value and accuracy value of ResNet’s training set and validation set. The second line represents the loss function value and accuracy value of the training set and validation set of the proposed method. Through comparison, it can be found that the method proposed in this paper has faster and more stable convergence speed and higher accuracy.

**FIGURE 7:**
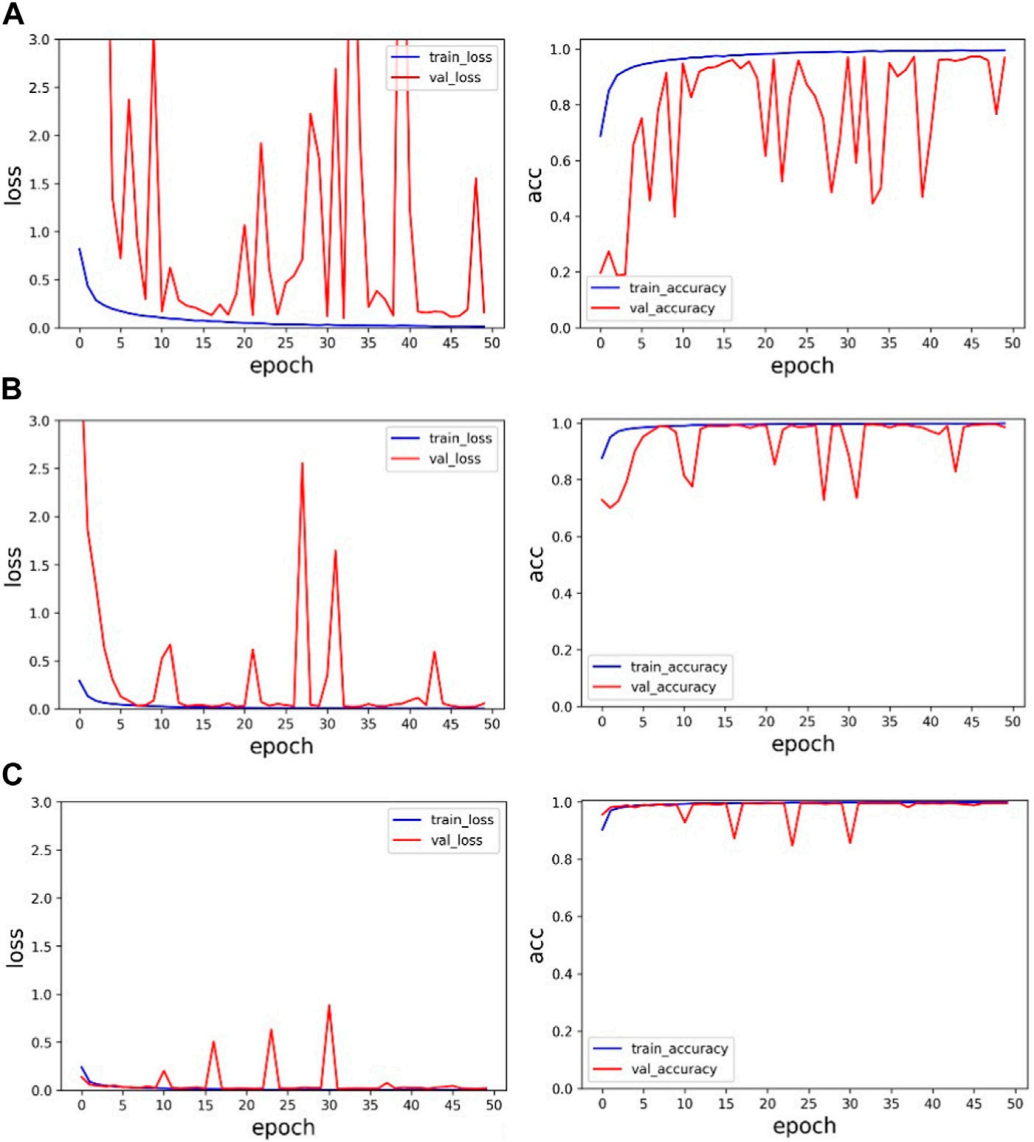
Single-channel and multi-channel experimental results: **(A)** is the experimental result of V5 channel, **(B)** is the experimental result of MLII, and **(C)** is the experimental result of multi-channel data.

Secondly, in order to verify the effectiveness of multi-channel data fusion for classification tasks, on the basis of the same model, the single-channel data (V5 and MLII) and the multi-channel fusion data were trained respectively, and the experimental results were shown in Table 4 and Figure 7. As shown from Table 4, the validation set accuracy of V5 channel, MLII channel and the proposed method is 96.84%, 98.54% and 99.60%, respectively, and the verification set accuracy of the proposed method is the highest, and the loss function value is also the lowest. Experimental results show that the dual-channel data is conducive to improving the accuracy of classification, and the proposed method can make full use of the dual-channel image information to achieve higher recognition accuracy. In Figure 7A represents the loss function value and ac-curacy value of training set and validation set under V5 channel. (b) Represents the loss function value and accuracy value of training set and validation set under MLII channel. (c) Represents the loss function value and accuracy value of training set and validation set under multi-channel fusion data. As can be seen from the figure, the value of the loss function after the fusion of two-channel data converges faster and is more stable. Its accuracy rate is also stable. Therefore, the proposed method can effectively extract the information of multi-channel ECG images, improve the accuracy of ECG classification, and the classification performance is optimal.

**TABLE 4:** The loss value and acc of the training and validation sets.

Methods	Train_loss	Val_loss	Train_acc	Val_acc
V5	0.01	0.15	99.51	96.84
MLII	0.002	0.05	99.91	98.54
Proposed	0.003	0.02	99.91	99.60

**FIGURE 8:**
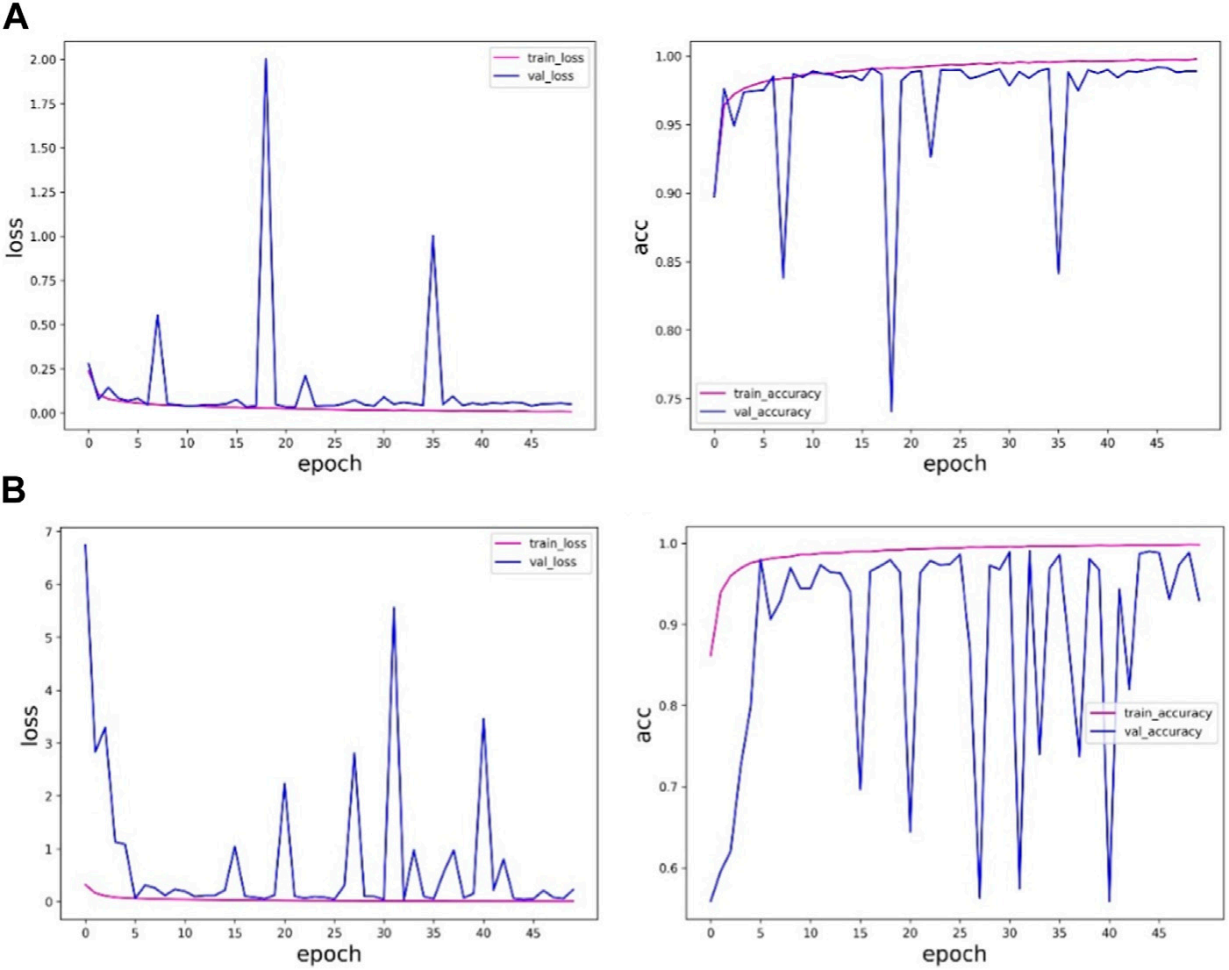
ResNet of the sudden cardiac death holter dataset and the experimental results of the method in this paper: **(A)** is the experimental results of ResNet, and **(B)** is the experimental results of the method in this paper.

By training the sudden cardiac death holter dataset, the experimental results of ResNet network architecture and fusion multi-scale modules are shown in Table 5 and Figure 8.

**TABLE 5:** Loss function value and accuracy for the sudden cardiac death holter dataset.

Methods	Train_loss	Val_loss	Train_acc	Val_acc
ResNet	0.007	0.049	99.76	98.89
Proposed	0.006	0.056	99.82	98.84

Table 5 and Figure 8 shows that the accuracy of the method proposed in this paper on the verification set is slightly lower than the results obtained by the ResNet network, and in the training process, the ResNet method is more stable and less fluctuating, while the method proposed in this paper fluctuates more, and the results are more uncertain, so the results obtained by the ResNet network are more trustworthy. The method proposed in this paper is not as good as the ResNet method. The reason may be that the sudden cardiac death holter dataset is complex, there are fewer samples with labeled information in this data, and the experimental samples of individual patients are limited.

In addition to comparing the ResNet algorithm, we trained the Goolenet network using the same data set. The experimental results are shown in Table 6 and Table 7. Table 6 shows the comparison results under the MITBIH dataset, and the optimal results in the table have been bolded. Except for the Kappa index, other indexes are superior to the results obtained by other algorithms. Table 7 shows the comparison results of the Sudden cardiac death holter dataset, and the optimal results in the table have been bolded. It can be seen that the results obtained by the ResNet algorithm are better. The reason may be that the dynamic ECG data set of sudden cardiac death is complex, and there are fewer samples with labeled information in the data, and the experimental samples of individual patients are limited. Bold fonts in Table 6 and 7 indicate the optimal experimental results.

**TABLE 6:** The comparison results of the algorithms under the MITBIH dataset.

Methods	Precision	Sensitivity	F1 score	Kappa
Goolenet	97.25	96.94	97.06	0.96
ResNet	97.74	97.58	97.62	0.96
Proposed	**98.25**	**98.38**	**98.33**	**0.97**

**TABLE 7:** Comparison results of algorithms under Sudden cardiac death holter dataset.

Methods	Precision	Sensitivity	F1 score	Kappa
Goolenet	99.18	**99.78**	99.47	0.99
ResNet	**99.63**	99.18	**99.48**	**1.0**
Proposed	99.34	99.16	99.24	**1.0**

To further verify the effectiveness of the proposed method, the results of some other ECG classification methods in recent years were compared under the same data set, as shown in Table 8. From the experimental results and Table 8, it can be concluded that this paper has obtained better results compared with other methods. Moreover, when adding multi-scale modules to the ResNet network, the effect is superior to that of the ResNet network, thus verifying that the proposed method is effective.

**TABLE 8:** This is a table. Tables should be placed in the main text near to the first time they are cited.

Author	Year	Accuracy (%)
Atal and Singh (2020)	2020	93.19
Xu and Liu (2020)	2019	98.60
Izci et al. (2019)	2019	97.42
Ullah et al. (2021)	2021	99.02
Wang et al. (2021)	2021	98.74
Proposed Method	2022	99.60

## 4 Discussion

t-SEN (t-Distributed Stochastic Neighbor Embedding) (Li et al., 2020) was proposed in 2008. The t-SEN feature is a better demonstration of experimental results. When there are many classifications, t-SEN can concisely demonstrate the classification performance of the classification model. As a result, we are able to more intuitively analyze the performance and quality of classification models through t-SEN visualization. The confusion matrix is used to summarize the predicted categories and the real categories in the form of a matrix.

**FIGURE 9:**
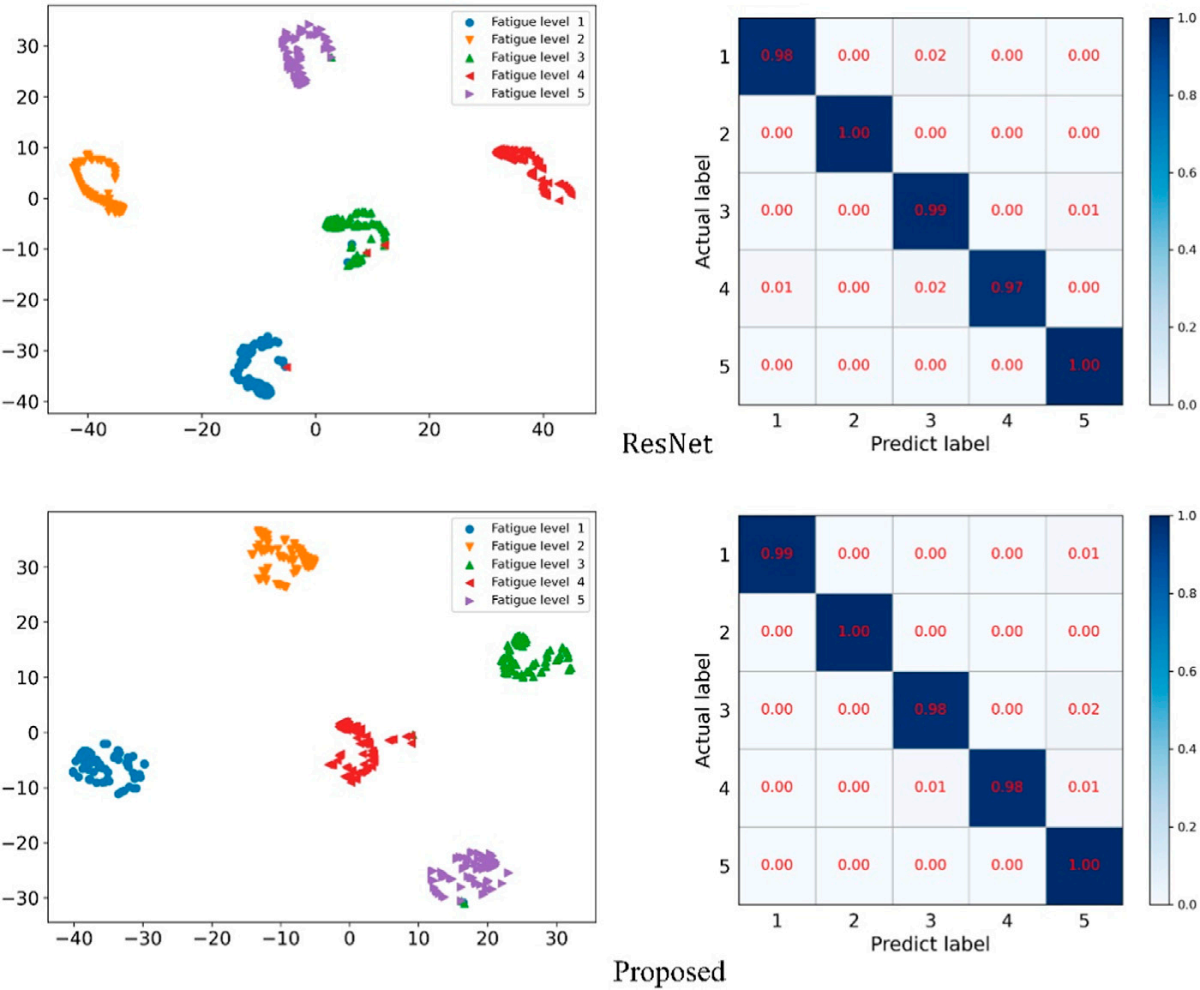
ResNet and the t-SNE and confusion matrix diagram of the method presented herein.

**FIGURE 10:**
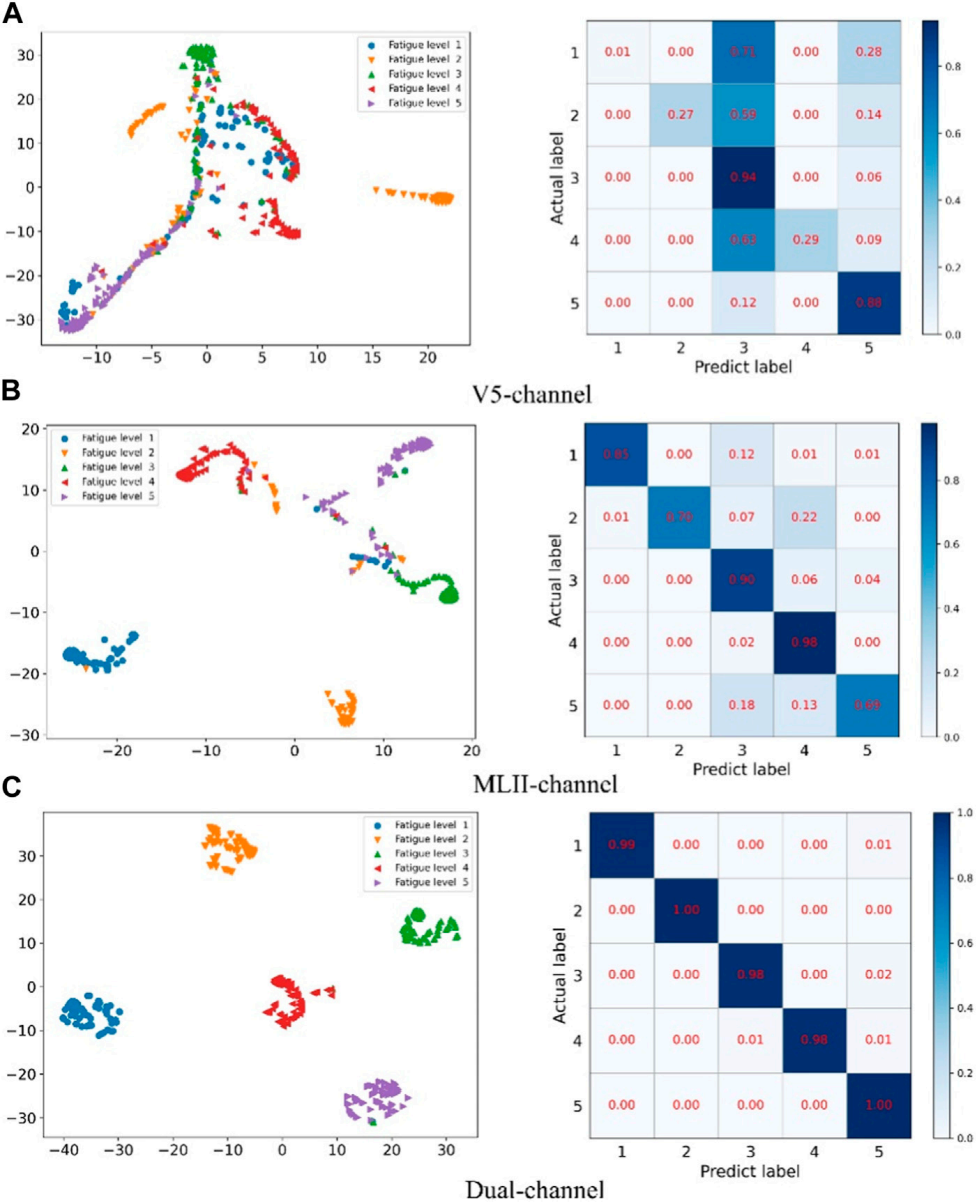
Confusion matrix and cluster analysis results for single-channel and multi-channel data fusion.

We use these two methods to visualize ResNet and the methods proposed in this paper, as shown in Figure 9 and Figure 10. Figure 9 compares the ECG image classification effect of ResNet and the proposed method, although both methods can achieve the 5-classification task of the ECG graph, but the proposed method in this paper has a lower error rate than ResNet on the t-SNE diagram. And in the confusion matrix, the recognition accuracy of both label 1 and label 4 is improved by 1%.

Figure 10 compares the confusion matrix and t-SNE plot of single-channel data and multi-channel data fusion. Among them A represents the V5 channel, B represents the MLII channel, and C represents multi-channel data fusion. The method proposed in this paper has clear clustering boundaries and the experimental data can be accurately divided into 5 categories. However, the classification results of single-channel data are very confusing and cannot effectively distinguish the categories of data.

**FIGURE 11:**
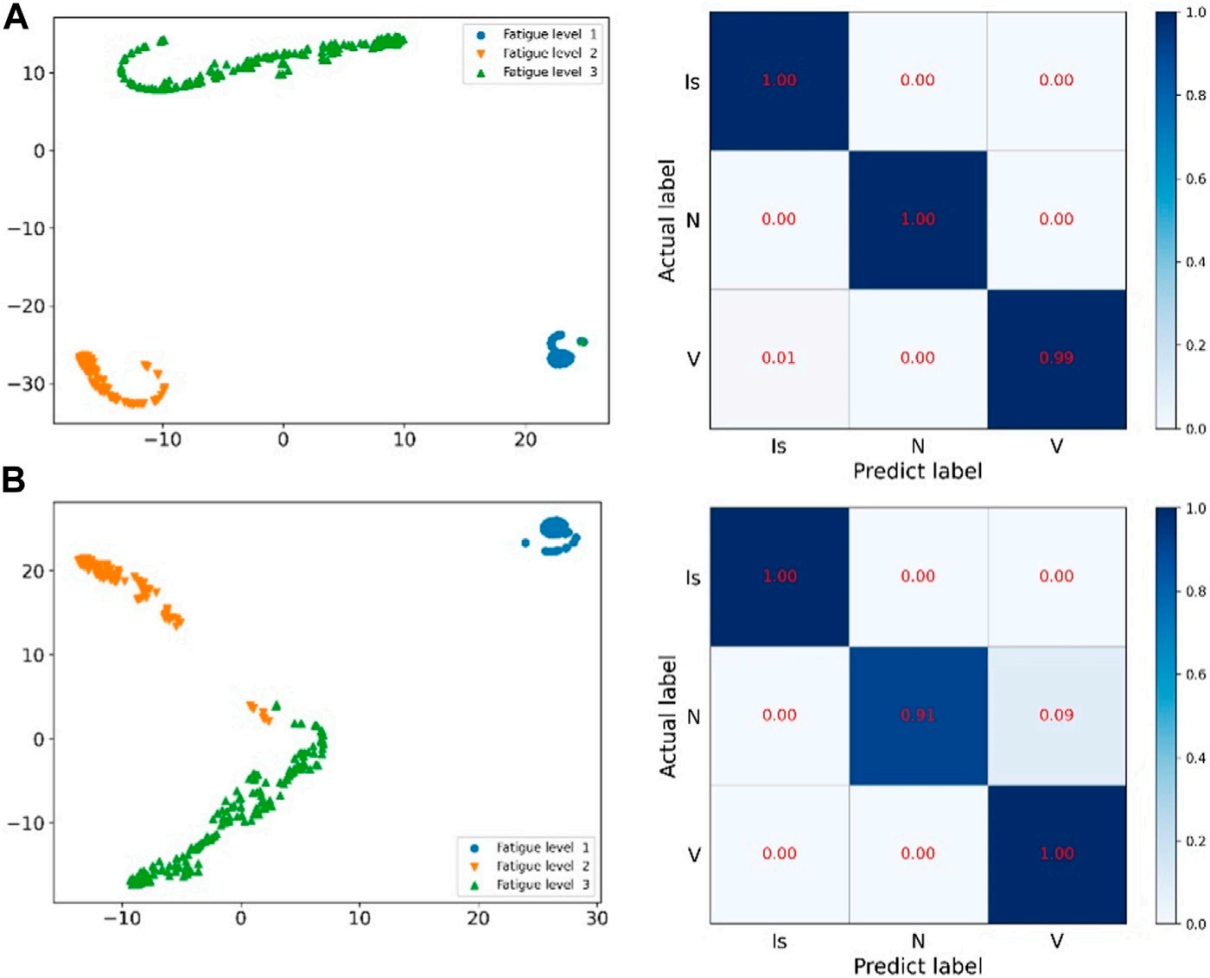
ResNet of the sudden cardiac death holter dataset and the prediction results of this method: **(A)** is the network of ResNet, **(B)** is the method of this paper.

**FIGURE 12:**
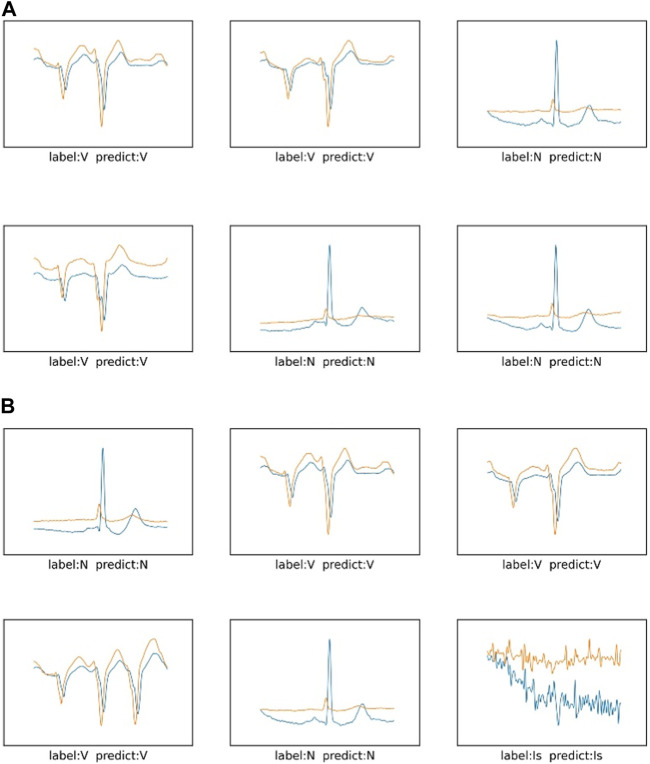
The prediction results of three categories in the sudden cardiac death holter dataset: **(A)** is the network of ResNet, **(B)** is the method of this paper.

The prediction effect of the network model is shown in Figure 11 and Figure 12 The prediction accuracy of the ResNet method on the three categories of I, N and V are 100%, 100% and 99%, respectively. However, the accuracy rate of the method proposed in this paper is 100%, 91% and 100% respectively on these three categories. The prediction accuracy rate of the method proposed in this paper is low for normal categories, and 9% of them are misclassified as V category. It is difficult for the proposed method to distinguish the normal category from the V category. In Figure 12, you can more intuitively see the prediction results of the ResNet method and the method proposed in this paper under the three categories. The N category and the V category are similar in some features. The method proposed in this paper is insufficient for feature learning of N and V categories. It is difficult to distinguish the N category from the V category when the two categories are similar.

Through the visualization of the experimental results, in the MITBIH dataset, compared with the simple residual network, the method proposed in this paper is significantly better than the comparison experiment. It can be seen that the multi-scale module has an effect on the ECG data of the MITBIH data set. The feature learning is better, the features of the data can be effectively extracted, and various ECG features can be accurately identified. In the sudden cardiac death holter dataset, the method proposed in this paper has a low classification accuracy for category N, confuses category N and category V, and classifies category N as category V. It can be seen that in the more complex sudden cardiac death holter dataset, the model proposed in this paper is not sufficient to accurately classify categories with similar characteristics. The method proposed in this paper needs to enhance the accuracy of data classification with subtle differences in ECG features.

## 5 Conclusion

In this paper, we propose an arrhythmia classification method based on multi-scale residual neural network and multi-channel data fusion. Considering that the information hidden in different lead ECG data is crucial for identifying arrhythmia diseases, we fused multi-channel ECG signals. In the process of ECG signal recognition, the residual neural network incorporates multi-scale modules, and uses the data after channel fusion to train the multi-scale residual neural network. After continuous optimization, the model can automatically extract image features from ECG images for arrhythmia classification with an accuracy rate of 99.60%. In addition, we also conduct experimental verification on single-channel data, and the accuracy rates reach 96.84% and 98.54%, respectively. Experimental results show that this method has better classification performance and is more conducive to the classification of arrhythmia. In future work, we will continue to develop portable wearable devices to diagnose more real-time samples.

## Data Availability

Publicly available datasets were analyzed in this study. This data can be found here: https://physionet.org/content/sddb/1.0.0/; https://physionet.org/content/sddb/1.0.0/.
